# Sustainment of Tobacco Use Treatment Programs Across National Cancer Institute–Designated Cancer Centers

**DOI:** 10.1002/cam4.71424

**Published:** 2025-11-26

**Authors:** Ramzi G. Salloum, Magda Montague, Mara Minion, Jennifer H. LeLaurin, Ji‐Hyun Lee, Edmond Ramly, Gonghao Liu, Miranda Reid, Carma L. Bylund, Danielle McCarthy, Donna Shelley, Jamie S. Ostroff, Graham W. Warren

**Affiliations:** ^1^ University of Florida College of Medicine Gainesville Florida USA; ^2^ University of Florida Health Cancer Center Gainesville Florida USA; ^3^ University of Wisconsin Center for Tobacco Research and Intervention Madison Wisconsin USA; ^4^ Indiana University School of Public Health Bloomington Indiana USA; ^5^ New York University School of Global Public Health New York New York USA; ^6^ Memorial Sloan Kettering Cancer Center New York New York USA; ^7^ University of Kentucky College of Medicine Lexington Kentucky USA

**Keywords:** implementation science, sustainability, sustainment, tobacco use treatment

## Abstract

**Background:**

Though tobacco use treatment (TUT) after a cancer diagnosis can improve cancer treatment outcomes and survival, delivery of evidence‐based TUT remains underutilized in cancer care. The National Cancer Institute (NCI) Cancer Center Cessation Initiative (C3I) implemented TUT across 52 NCI‐Designated Cancer Centers, but there is little information on its long‐term sustainment. This study assesses TUT sustainment beyond initial implementation in C3I.

**Methods:**

A web‐based survey across 52 C3I centers was conducted during the sustainment phase (2023–2024) following NCI C3I funding. The surveys assessed program funding and the sustainment of the overall program, program components and practices, assessment of implementation and patient outcomes, partnerships, and program scale‐out across settings. The survey data were analyzed using descriptive statistics.

**Results:**

Among 47 responding sites (90% response rate), 83% reported continued TUT activity after NCI funding ended with annual operating budgets between $100,000 and $250,000. Most sites (78.7%) reported some institutional support, while few relied on fee‐for‐service reimbursement (27.7%), bundled payments (8.1%), or support from grants (27.7%) and philanthropic donations (21.3%). Key program components including electronic health record modifications, outcomes reporting, and staff training were largely maintained, with nearly all (46) sites continuing to screen for tobacco use and refer patients to TUT. Perceived program partnerships were strongest with clinicians and departmental leadership, and some programs were scaled out to primary care and other specialties.

**Conclusions:**

Results confirm that most cancer centers sustained key TUT program functions and partnerships with some increasing TUT delivery across larger cancer treatment settings.

## 1 Background

Approximately 10%–15% of cancer patients and survivors continue smoking after their cancer diagnosis [1, 2, 3]. Continued smoking by cancer patients and survivors increases overall and cancer‐related mortality, risk for second primary cancer, and is associated with increased toxicity from cancer treatment [4, 5, 6, 7, 8], The adverse effects of smoking are associated with significant added costs to cancer treatment [9, 10]. Quitting smoking at or following a cancer diagnosis significantly improves survival and cancer treatment outcomes [11, 12, 13, 14, 15, 16, 17, 18]. Importantly, quitting smoking after a cancer diagnosis results in optimal survival benefits, with an approximate 4‐year improved median survival for patients who quit within 6 months following a diagnosis [11].

Most cancer organizations provide clear advocacy and guidelines for tobacco use treatment (TUT) as a core component of high‐quality cancer care [7], but sustainable delivery of evidence‐based care remains elusive. The prevalence of continued tobacco use among cancer patients and survivors is high [2, 3, 19]. Surveys of oncology providers demonstrate that while most ask about tobacco use and advise patients to quit, only approximately one third regularly assist patients with quitting [20, 21]. Moreover, several years ago, assessments of cancer centers demonstrated that most cancer care settings did not consistently provide effective TUT to their patients [22]. These practice gaps may explain why at least half of cancer patients and survivors who report smoking at diagnosis continue to smoke after their cancer diagnosis [2, 23, 24, 25]. Despite the clinical benefits of tobacco cessation in cancer care, multilevel challenges contribute to low rates of TUT including lack of time, lack of training, lack of resources, and perceptions by patients and clinicians associated with stigma, emotional distress, and fatalism [1, 25, 26, 27, 28, 29, 30, 31, 32].

In response to this gap in the delivery of evidence‐based TUT, the National Cancer Institute (NCI) convened a conference in 2009 [33] and launched the Cancer Center Cessation Initiative (C3I) in 2017 to support the greater implementation of TUT across 52 NCI‐Designated Cancer Centers over three cohorts (2017–2019, 2018–2020, 2020–2021) [34]. The C3I recognized the need to address multilevel challenges, such as the method of tobacco screening, integration into clinical workflow across multiple clinical settings, use of information technology, and systematic outcomes reporting. Each C3I funded center could implement any combination of evidence‐based TUT approaches that best fit with their resources and patient populations and were required to commit to sustaining their programs for a minimum of 3 years beyond initial NCI funding. The C3I significantly increased the implementation of TUT across funded sites including improved TUT screening, referral rates, quit rates, training, clinical workflows, electronic health record (EHR) documentation, and organizational engagement [35, 36]. The objective of this paper is to report on the sustainment of TUT programs 2–4 years following the extramurally funded C3I implementation phase.

## 2 Methods

The methodologic approaches and implementation infrastructure provided by C3I have been published elsewhere [35, 36]. In brief, during the C3I implementation phase (2017–2021), the Coordinating Center at the University of Wisconsin Madison administered a web‐based survey to the participating centers every 6 months using Qualtrics Software (Provo, UT). Participating centers were required to complete these assessments. Surveys were disseminated to the principal investigator (PI) of the C3I supplement award and/or TUT program lead(s) and assessed center characteristics and implementation processes and outcomes. The Coordinating Center facilitated access to the prospective data collected during the implementation phase. For this sustainment study, the research team administered an additional voluntary Qualtrics survey to C3I site leaders between November 2023 and April 2024. The sustainment phase survey assessed sustainment outcomes, including whether the program was still operating and reasons for discontinuing operations (if applicable). The survey also assessed TUT program budget and funding sources because the transition from temporary to permanent funding and the inclusion of an annual program budget have been identified as critical for institutionalization and sustainability [37]. We followed guidance by Scheirer and Dearing on the evaluation of program sustainment [38] that identify the following sustainment outcomes: (1) sustained overall program, (2) sustained program components and practices (started during implementation), (3) sustained assessment of program implementation and patient outcomes, (4) sustained partnerships (developed during the funded program), and (5) scaled program to other settings.

All C3I sites were administered full surveys, regardless of operational status. If they were no longer operating, sites were asked to report on their last operational year. Respondents received an incentive of $199 for completing the survey. The survey is available in the Appendix S1 section. The study was deemed exempt by the University of Florida Institutional Review Board and informed consent was not required.

### 2.1 Sustainment Outcomes

#### 2.1.1 Sustained Overall Program

Centers were asked to report whether their programs were still operating at the time of the survey. Response options included: (1) yes, our program still offers TUT services; (2) no, and TUT services are offered via another entity; and (3) no, and tobacco use treatment services are no longer available. For programs that were reported to be no longer operating, the centers were asked to report when their program stopped operations (month and year) and the primary reasons it stopped operating (sites could report multiple reasons): (1) financial constraints/loss of funding; (2) lack of leadership buy‐in/institutional commitment; (3) lack of provider buy‐in; (4) lack of referrals to the program; (5) lack of patient interest; (6) lower numbers of patients who smoke; (7) lack of reporting on outcomes; (8) loss of staff/staff turnover; and (9) loss of clinic champions/key personnel.

#### 2.1.2 Sustained Program Components and Practices

Centers were asked to indicate the status of the following program activities and components that were previously assessed by the C3I Coordinating Center in the implementation phase: (1) cessation medications provided or covered by the program; (2) staff education and training about the program; (3) technology services (e.g., interactive voice response); (4) equipment (e.g., computers); (5) office space; (6) resources related to EHR modifications including access to EHR IT experts; (7) patient recruitment support; (8) community outreach and engagement; (9) marketing and communications; and (10) reporting to monitor program outcomes. Response options were: never implemented, no longer maintained, decreasing, increasing, or maintaining.

#### 2.1.3 Sustained Assessment of Program Implementation and Patient Outcomes

Centers were asked to report on current monitoring of the following program metrics: tobacco use status, program referrals, reach, and effectiveness. For each of these metrics, centers were asked whether the program: (1) continues to assess using the C3I definition; (2) assesses using a different format; or (3) does not currently assess.

#### 2.1.4 Sustained Partnerships

Centers were asked to indicate the degree to which they perceive each of the following partners to be engaged in the program: (1) patients and caregiver advisory groups; (2) community oncology network leadership; (3) pharmacy; (4) clinicians and staff who implement the program; (5) department in which the program is housed; (6) cancer center leadership; (7) clinical center of excellence (i.e., clinical entity that oversees cancer care); (8) clinical council (i.e., entity that translates national guidelines into local practice); (9) chief executive officer of the health system; (10) chief financial officer; (11) chief medical officer; (12) chief information officer; (13) chief nursing officer; (14) chief quality officer; (15) academic dean (e.g., of the medical school); and (16) health system board of directors.

#### 2.1.5 Scaled Program to Other Settings

Centers were asked to report on the number of sites that piloted the program (in the first year of C3I funding), the number of sites in which the program is currently implemented, and the number of sites in which the program is expected to be in 1 year from the time of the survey. To assess change in the number of settings providing TUT in each phase as a measure of scaling TUT services, centers were asked to report on these measures for the following settings separately: medical oncology, radiation oncology, surgical oncology, primary care, other non‐oncology specialty clinics, and affiliated clinics and health systems (e.g., regional hospitals).

##### 2.1.5.1 Program Budget and Funding Sources

Centers were asked to report the highest annual operational budget for their program in the past 5 years, the annual operational budget in 2023 (or the program's final year of operations), and how much additional funding would be needed to support an optimal program. Response options for all financial questions were: (1) < $50,000; (2) $50,000–$100,000; (3) $100,000–$250,000; (4) $250,000–$500,000; and (5) > $500,000. Centers were then asked to estimate the percentage of funding received from the following sources for 2023 or the final year the program operated: (1) fee for service (clinical revenue) reimbursement; (2) bundled or episode‐based payments; (3) institutional support; (4) grant funding; and (5) charitable contributions.

##### 2.1.5.2 Analysis and Presentation of Results

Qualtrics survey data were converted to R data types. Our unit of analysis was the C3I center. All collected data, including characteristics of participating sites were summarized using descriptive statistics along with graphical illustrations. Descriptive statistics (i.e., frequency/proportion for discrete data and mean/standard deviation (SD) for continuous data) were summarized for each outcome. The study was deemed to be nonhuman research by the University of Florida Institutional Review Board as no patient‐level data were used.

## 3 Results

### 3.1 Cancer Center Characteristics

Among the 52 NCI‐Designated Cancer Centers that participated in C3I, 84.6% were standalone cancer centers and 15.4% were matrix cancer centers (Table 1). All but two cancer centers (96.2%) had the Comprehensive designation. One quarter (25%) of the centers were in the Northeast, 19.2% in the Midwest, 32.7% in the South, and 23.1% in the West. The cancer centers treated a mean of 22,724 (SD, 30,342) patients annually, including a 6‐month mean smoking prevalence of 12.7% (SD = 13.4%). Among these centers, 21.1% reported losing the sole principal investigator (PI) of their C3I award or both PIs (when applicable) and 13.5% reported losing one of their dual PIs.

**TABLE 1:** Characteristics of the 52 NCI‐Designated Cancer Centers participating in the Cancer Center Cessation Initiative (C3I), 2023–2024.

Cancer center characteristic	*N* = 52
Cancer Center Structure, *N* (%)	
Standalone cancer center	44 (84.6%)
Matrix cancer center	8 (15.4%)
NCI designation type, *N* (%)	
Comprehensive cancer center	50 (96.2%)
Clinical cancer center	2 (3.8%)
US Census region, *N* (%)	
Northeast	13 (25%)
Midwest	10 (19.2%)
South	17 (32.7%)
West	12 (23.1%)
Cancer center size (annual patient volume)^a^, ^c^	
Median (min, max)	12,862 (424, 151,377)
Mean (SD)	22,724 (30,342)
No. of patients who smoke (at 6 months)^a^, ^c^	
Median (min, max)	956 (6, 7665)
Mean (SD)	1766 (1885)
Center smoking prevalence (at 6 months)^a^, ^b^, ^c^	
Median (min, max)	9.5 (0.3, 66.7)
Mean (SD)	12.7 (13.4)
Principal investigator (PI) departure, *N* (%)	
Loss of sole or both PIs	11 (21.2%)
Loss of one of the dual PIs	7 (13.5%)

*Note:* Means and medians reflect occurrence across participating C3I centers.

^a^ One cancer center did not respond to these questions in the implementation phase surveys.

^b^ Defined as the number of patients who smoke among adult patients (18 years and older) with a visit during the reporting period.

^c^ Data collected between July–December 2022 by the C3I Coordinating Center; for sites that did not provide data for this time period, or the most recently available 6‐month reporting period was used instead.

### 3.2 Sustainment Outcomes

#### 3.2.1 Sustained Overall Program

The survey had a 90% response rate (47 of 52 sites). Among respondents, 39 (83%) sites continued to offer TUT after NCI funding for the initiative had ended. Consequently, the operating status of the 5 non‐responding sites was not documented. Among the sites still operational, 97.4% continued to screen patients for tobacco use and 94.9% continued to refer patients to TUT.

#### 3.2.2 Sustained Program Components and Practices

**FIGURE 1:**
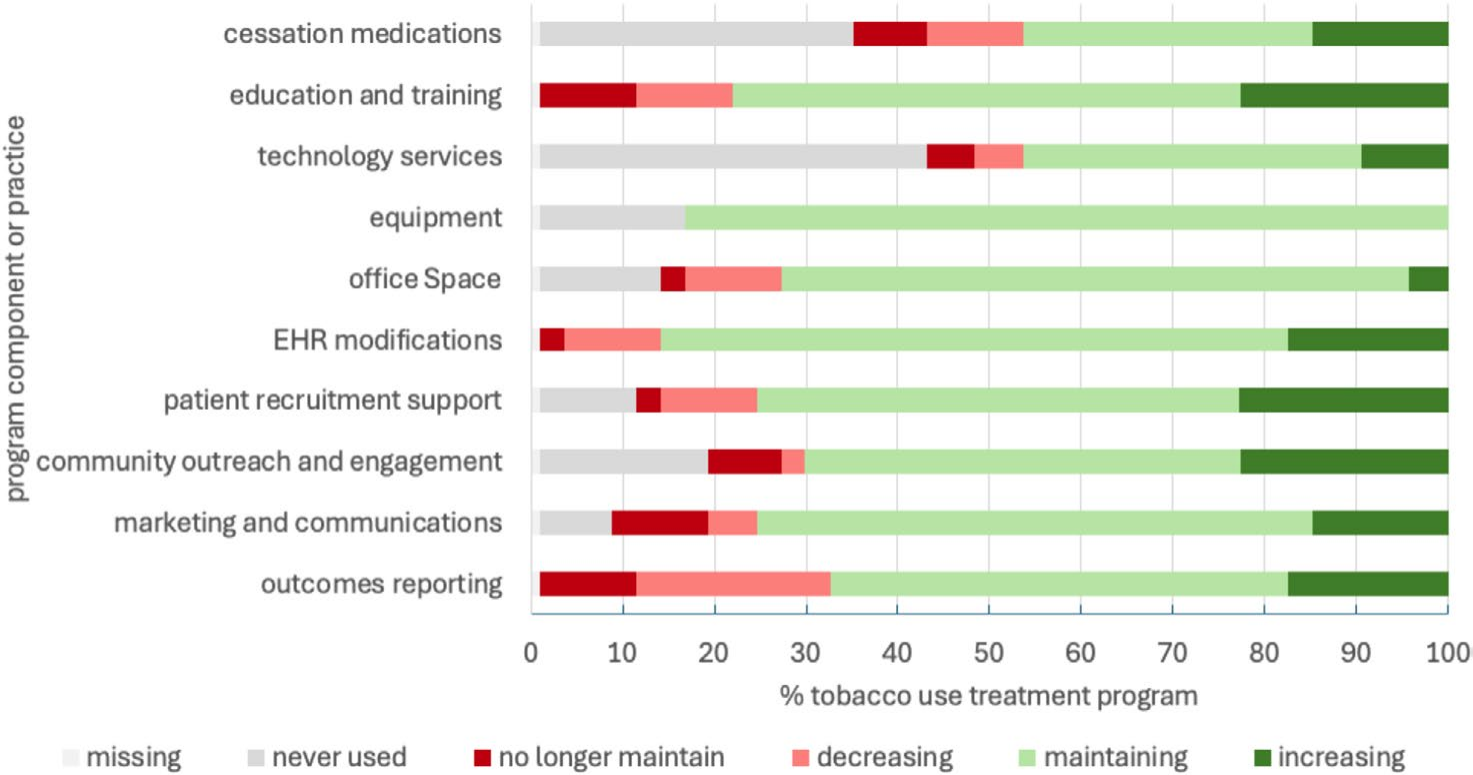
Maintenance of tobacco use treatment program components and practices.

EHR modifications were maintained at 86.8% of operating sites, 68.4% of sites continue reporting outcomes, 79% maintained education and training, 76.3% continued marketing and communications activities, and 71.1% still engaged in community outreach and engagement (Figure 1). Alternatively, 10.5% of sites no longer maintained marketing and communication activities as well as education and training activities that they had started during the implementation phase. Likewise, 10.5% of sites no longer maintained outcomes reporting.

#### 3.2.3 Sustained Assessment of Program Implementation and Patient Outcomes

Additionally, 87.2% of sites have maintained the ability to assess TUT reach and 79.5% have maintained the ability to assess TUT effectiveness.

#### 3.2.4 Sustained Partnerships

**FIGURE 2:**
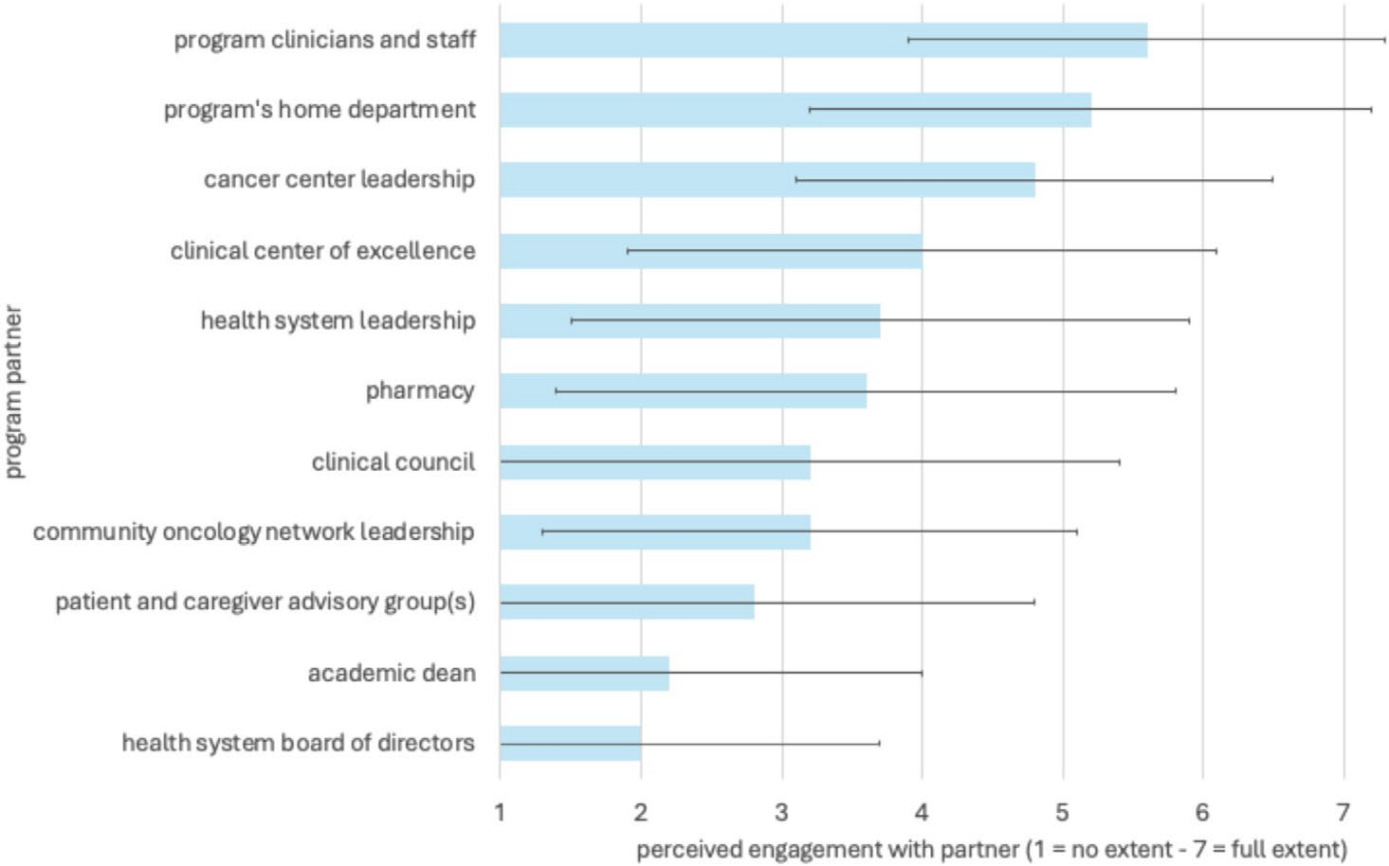
Perceived maintenance of partnerships by the tobacco use treatment programs, mean (SD).

In terms of perceived engagement with partners (Figure 2), the highest average score was for program clinicians and staff (mean [SD] = 5.6 [1.7], scale = 1.0–7.0), followed by the program's home department (5.2 [2.0]), and cancer center leadership (4.8 [1.7]). The lowest average score was for the health system board of directors (2.0 [1.7]) followed by the academic dean (2.2 [1.8]).

#### 3.2.5 Scaled Program to Other Settings

**FIGURE 3:**
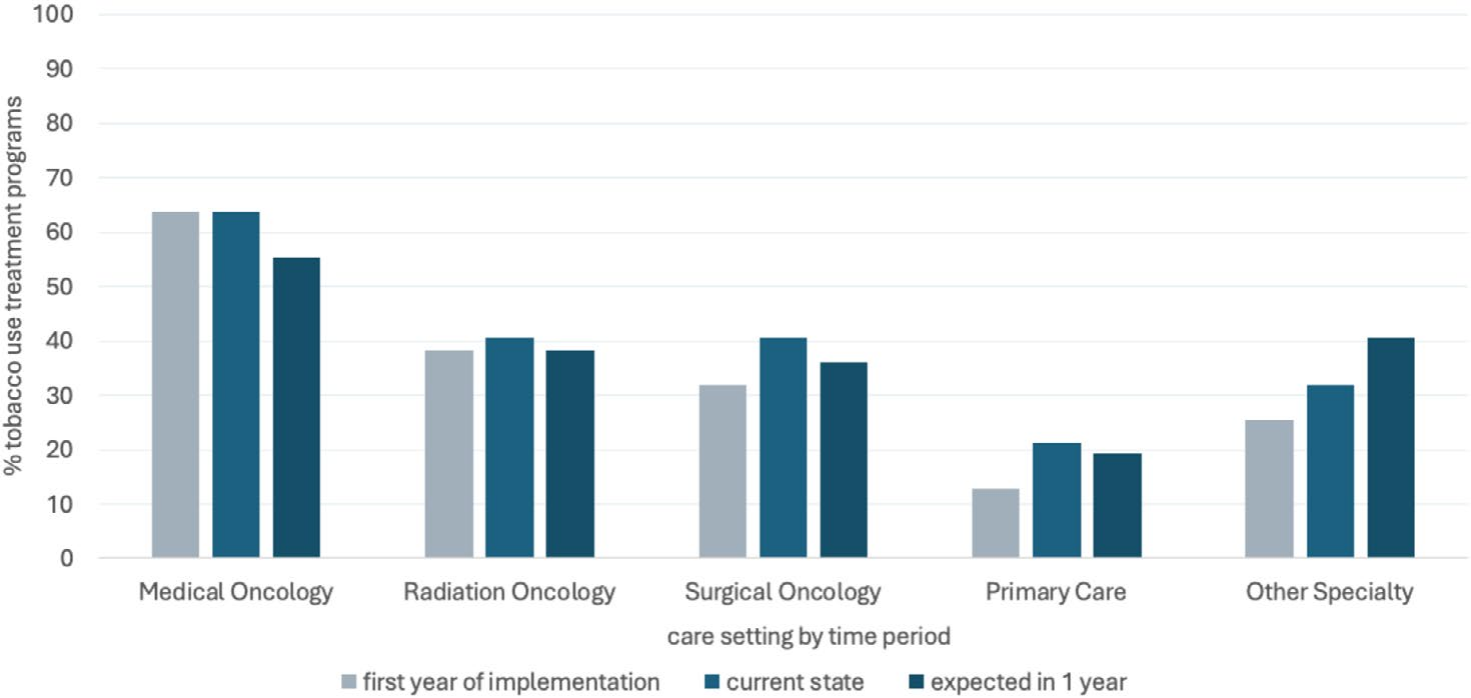
Scaling of tobacco use treatment programs across care settings by time period.

During the implementation phase, 63.8% of sites responding to the survey had offered TUT services in their medical oncology sites, compared with 38.3% in radiation oncology and 31.9% in surgical oncology. These rates were largely maintained for medical oncology and radiation oncology at the time of reporting, while the rate increased to 40.4% for surgical oncology, to 21.3% for primary care, and to 31.9% for other specialties. During the sustainment phase, 21.3% had scaled‐up services to primary care patient populations, and 31.9% had extended services to other specialties (Figure 3).

### 3.3 Program Budget and Funding Sources

**FIGURE 4:**
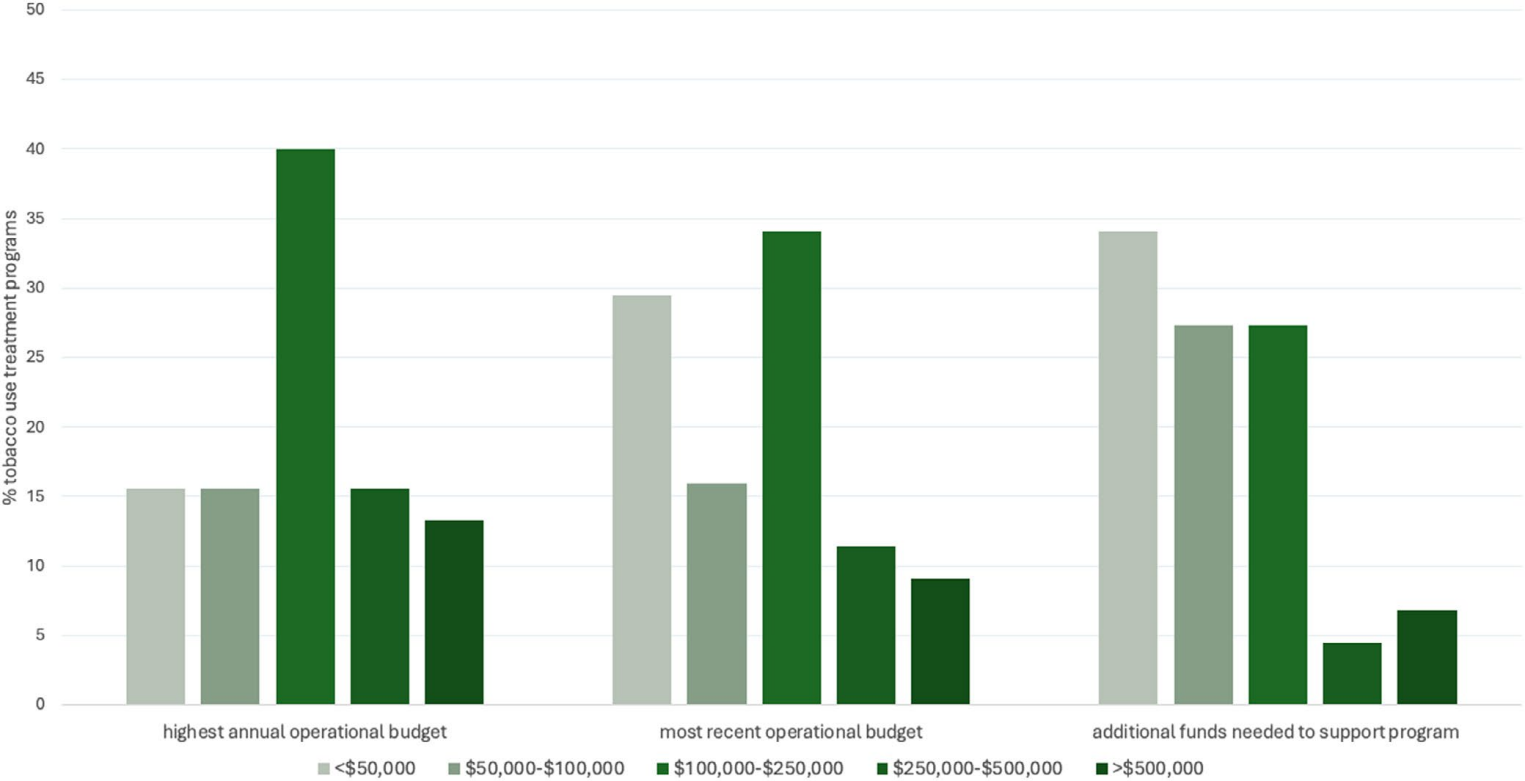
Tobacco use treatment program funding: Proportions of highest annual operational budget, most recent annual operational budget, and additional funding needed to support program. Most recent operational budget was for 2023 for operational programs and last operational year for nonoperational programs.

**FIGURE 5:**
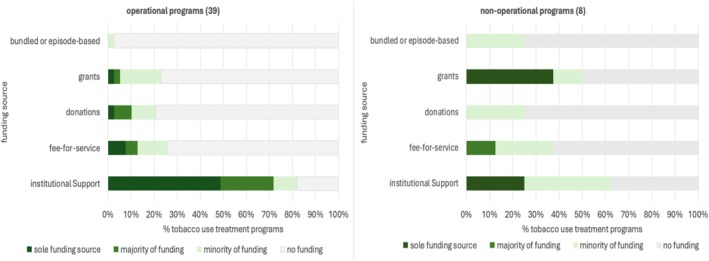
Tobacco use treatment program funding source by operational status: Operational programs (left panel) and nonoperational programs (right panel).

The most reported range for the highest annual operational budget was $100,000 to $250,000 (34.1% of sites) (Figure 4). The annual operational budget in 2023 (or the last year of operations) was $100,000–$250,000 among 34.1% of sites, followed by $50,000 among 29.5% of sites. When asked how much additional funds were needed to support an optimal program, 34.1% of sites indicated < $50,000 were required, followed by $50,000 to $100,000 and $100,000 to $250,000 (27.3% for each category). In terms of financing strategies (Figure 5), 82.1% of operating sites reported at least some institutional support for the program, compared with 62.5% for sites no longer operational (i.e., during the last year of operations). Only 25.6% of operating sites and 37.5% of nonoperating sites reported fee‐for‐service clinical revenue as a funding source. Charitable donations were among the funding sources in 20.5% of operating sites compared with 25% of nonoperating sites. Grants were among the funding sources in 23.1% of operating sites and 50% of nonoperating sites. Finally, bundled or episode‐based payments were used by only 2.6% of operating sites and two (25%) nonoperating sites.

## 4 Discussion

To our knowledge, this study is among the first to examine how evidence‐based TUT practices are sustained in cancer care settings on a national scale. The sustainment phase survey of the NCI‐Designated Cancer Centers that participated in C3I revealed that 82% of sites continued offering TUT after NCI funding ended. Most sites reported that they maintained core program components such as EHR modifications, outcomes reporting, and staff education, and nearly all continued screening patients for tobacco use and referring them to TUT. Engagement was strongest with program staff and departmental leadership, with less engagement among institutional leadership, patients, and community networks. Some programs were scaled up in other clinical settings, including primary care and other specialties. These encouraging findings are particularly noteworthy when compared to the prior literature noting gaps in the availability of TUT services across NCI‐designated cancer centers [22, 33].

The majority of sites reported operating on annual program budgets between $100,000 and $250,000, which was consistent with the level of support provided by C3I during the implementation phase. Institutional support was the most common financing strategy, while fee‐for‐service reimbursement was less frequently used and bundled payments were rarely used; grants and charitable donations played an even smaller role. Our survey data suggest that the majority of C3I programs have sustained their highest operational budget; however, the percentage of sites operating with a budget of below $50,000 doubled, suggesting sites are maintaining operations within a reduced scope. When asked how much more funding they would need (currently or during their last operational year), over 80% reported they would need less than $100,000/year in additional funding to optimize their programs. In the context of the cost of contemporary cancer treatment, these data suggest that a relatively small investment could sustain tobacco cessation interventions that have proven health and survival benefits, and reduce downstream costs associated with additional cancer treatments caused by continued smoking [4, 5, 7, 9, 10].

Medical procedures, coding, required resources, and relative value are defined by agencies such as the Centers for Medicare and Medicaid Services (CMS) resource‐based relative value scale (RVS) Update Committee (RUC) and the Relative Value Use (RVU) Committee [39, 40]. Reviews demonstrate diverse approaches to reimbursable TUT strategies [41, 42], but the survival‐based value of smoking cessation after a cancer diagnosis [9, 10, 11] may not currently receive value‐based support for billing or reimbursement comparable to value‐based support for traditional cancer treatment agents. Our findings demonstrate that greater diversification of funding sources and financial support from the cancer center or health system were both associated with program sustainment. These data support the need to consider comprehensive reassessment and assignment of financial support to sustain program activities and underscore the importance of strong institutional commitment to continue to provide evidence‐based TUT in the context of cancer care.

In alignment with improved financial support, data suggest that there is a relative paucity of engagement with senior institutional leadership and community outreach networks. Cancer center leadership should be engaged and aware of the need to allocate TUT resources to deliver evidence‐based care that directly improves patient health and survival. Leadership engagement has been consistently identified as one of the most influential factors in sustaining healthcare programs [43, 44], and communicating the value of the program and specifically articulating how TUT aligns with leadership priorities (e.g., patient safety, catchment area) may reduce the likelihood that necessary TUT program resources are reduced or eliminated. During the COVID‐19 pandemic, TUT services across the United States and Canada were adapted to telehealth, canceled or their staff were reassigned in part because executive leadership had to make rapid real‐world health system decisions to remain adaptable to changing healthcare needs [45, 46]. Efficient communication among health system leadership, clinical operations, and TUT program leadership could minimize a lack of awareness about the importance of TUT to improve cancer treatment outcomes as well as reduce readmissions, hospitalizations, healthcare costs, and resource utilization [4, 5, 7, 11, 47, 48]. Because TUT impacts cancer diagnosis, treatment, outcomes, as well as management of other health conditions, sustainable TUT almost certainly requires multidisciplinary communication across all levels of care and leadership.

There are several limitations to the results from this study. While these data are the largest report on the sustainment of TUT, or other behavioral health and addiction treatment, across 52 cancer care centers, they represent a cross‐sectional time point only, and the authors acknowledge that sustainability is a dynamic construct. These data may not represent the clinical needs, workflows, and implementation strategies for smaller community‐based cancer care sites. Results were collected during tectonic changes in local, regional, national, and international healthcare systems during the COVID‐19 pandemic which clearly affected TUT implementation and sustainability [45]. Results may have been significantly different without these unprecedented challenges to cancer care delivery. Some surveys were completed by investigators or staff that were not a part of the original C3I implementation phase, thereby posing a risk for subjective response bias. Finally, our survey did not include operational metrics such as changes in staffing, which are important measures of program sustainability and capacity.

The C3I is one of three large scale implementation initiatives for TUT in cancer care. Parallel activities by the Canadian Partnership Against Cancer (CPAC) increased access to evidence‐based TUT from 26% of ambulatory cancer centers across Canada in 2016 to 95% of centers by 2022 [49]. Importantly, whereas up‐front billing is a primary driver for sustainable medical services in the United States, long term healthcare savings are a significant driver and a central concept for dissemination across jurisdictions in Canada [50]. Another effort by the American College of Surgeons (ACOS) Commission on Cancer (CoC), resulted in the Just ASK and Beyond ASK implementation initiatives to improve identification of tobacco use and assistance with quitting [51, 52]. The CoC implementation initiatives had the largest absolute reach for addressing TUT, touching approximately one third of newly diagnosed cancer patients across the United States. However, assessments for CoC were limited due to a shorter implementation timeframe. Collectively, the C3I, CPAC, and CoC initiatives have all demonstrated that TUT implementation is feasible. Findings from this work advance the field and provide a foundation for future research to identify factors that influence sustainability, and test strategies that optimize sustainability. In addition to studying the influence of variation in operating budget, other contextual variables such as organizational commitment, capacity and culture, as well as specific determinants of effective partnerships, would be helpful in guiding cancer centers to implement and sustain their TUT programs. Moreover, mandated requirements for NCI designation or accreditation by organizations such as CoC may have significant effects on the implementation and sustainability of TUT services across cancer care.

## Author Contributions

**Ramzi G. Salloum:** conceptualization (equal), funding acquisition (equal), methodology (equal), project administration (equal), resources (equal), supervision (equal), writing – original draft (equal), writing – review and editing (equal). **Magda Montague:** data curation (equal), project administration (equal), supervision (equal), writing – review and editing (equal). **Mara Minion:** methodology (equal), project administration (equal), writing – review and editing (equal). **Jennifer H. LeLaurin:** methodology (equal), writing – review and editing (equal). **Ji‐Hyun Lee:** formal analysis (equal), methodology (equal), supervision (equal), writing – review and editing (equal). **Edmond Ramly:** methodology (equal), writing – review and editing (equal). **Gonghao Liu:** formal analysis (equal), methodology (equal), writing – review and editing (equal). **Miranda Reid:** methodology (equal), writing – review and editing (equal). **Carma L. Bylund:** methodology (equal), writing – review and editing (equal). **Danielle McCarthy:** project administration (equal), writing – review and editing (equal). **Donna Shelley:** conceptualization (equal), investigation (equal), methodology (equal), writing – review and editing (equal). **Jamie S. Ostroff:** conceptualization (equal), investigation (equal), methodology (equal), writing – review and editing (equal). **Graham W. Warren:** conceptualization (equal), funding acquisition (equal), investigation (equal), methodology (equal), writing – original draft (equal), writing – review and editing (equal).

## Funding

This study was primarily funded by a grant from the NCI (R01CA279890).

## Conflicts of Interest

The authors declare no conflicts of interest.

## Supporting information


**Appendix S1:** cam471424‐sup‐0001‐AppendixS1.docx.

## Data Availability

The datasets analyzed during the study are not publicly available due to the sensitive nature of some data. The sustainment survey dataset is available from the corresponding author upon reasonable request. All other data are available from the C3I coordinating center upon reasonable request.
