# Supplementary material for: Sustainment of Tobacco Use Treatment Programs Across National Cancer Institute–Designated Cancer Centers
Source: Cancer Med. 2025 Nov 26;14(22):e71424. doi: 10.1002/cam4.71424 (PMC12648423; doi:10.1002/cam4.71424)
Supplement: Supplementary file 1 — Appendix S1: cam471424‐sup‐0001‐AppendixS1.docx. [file CAM4-14-e71424-s001.docx]

**Supplementary Materials to “Sustainability of Tobacco Use Treatment Programs Across National Cancer Institute-Designated Cancer Centers”.**

**Cancer Center Cessation Initiative (C3I) Sustainability Survey**

## **PROGRAM STATUS**

1. Is your program still operating?
   1. Yes, our program still offers tobacco treatment services
   2. No, but tobacco treatment services are offered via another entity.
   3. No, and tobacco treatment services are no longer available

*Yes:* **Please answer all of the questions in this survey based on the current standing of the Tobacco Treatment program in the year 2023.**

*No:* 2. When did your Tobacco Treatment Program stop operations (please provide month and year if possible? _____________

1. What would you say are the primary reasons it stopped operating (Select 1-3 main reasons)
   1. Financial constraints/loss of funding
   2. Lack of leadership buy-in/institution commitment
   3. Lack of provider buy-in
   4. Lack of referrals to the program
   5. Lack of patient interest
   6. Lower numbers of patients who smoke
   7. Lack of reporting on outcomes
   8. Loss of staff/staff turnover
   9. Loss of the program leadership
   10. Loss of clinic champions/key personnel
   11. Other reason, please specify:_________

**Please answer all of the questions in the survey based on the last year your program was operational.**

## **DETERMINANTS**

**In the following questions, you will rate your Tobacco Treatment Program across a range of specific factors that affect sustainability. Please respond to as many items as possible. If you truly feel you are not able to answer an item, you may select “NA.” For each statement, select the number that best indicates the extent to which your practice has or does the following things.**

*Engaged Staff & Leadership: Having supportive frontline staff and management within the organization*

|  | **1- To little or no extent** | **2** | **3** | **4** | **5** | **6** | **7- To a very great extent** | **N/A** |
| --- | --- | --- | --- | --- | --- | --- | --- | --- |
| The tobacco treatment program engages leadership and staff throughout the process. |  |  |  |  |  |  |  |  |
| Clinical champions of the tobacco treatment program are recognized and respected. |  |  |  |  |  |  |  |  |
| The tobacco treatment program has engaged, ongoing champions. |  |  |  |  |  |  |  |  |
| The tobacco treatment program has a leadership team made of multi-professional partnerships. |  |  |  |  |  |  |  |  |
| The tobacco treatment program has team-based collaboration and infrastructure. |  |  |  |  |  |  |  |  |

*Engaged Stakeholders: Having external support and engagement for the practice.*

|  | **1- To little or no extent** | **2** | **3** | **4** | **5** | **6** | **7- To a very great extent** | **N/A** |
| --- | --- | --- | --- | --- | --- | --- | --- | --- |
| The tobacco treatment program engages the patient and family members as stakeholders. |  |  |  |  |  |  |  |  |
| There is respect for all stakeholders involved in the tobacco treatment program. |  |  |  |  |  |  |  |  |
| The tobacco treatment program is valued by a diverse set of stakeholders. |  |  |  |  |  |  |  |  |
| The tobacco treatment program engages other medical teams and community partnerships as appropriate. |  |  |  |  |  |  |  |  |
| The tobacco treatment program team has the ability to respond to stakeholder feedback about the program. |  |  |  |  |  |  |  |  |

*Monitoring & Evaluation: Assessing the practice to inform planning and document results*

|  | **1- To little or no extent** | **2** | **3** | **4** | **5** | **6** | **7- To a very great extent** | **N/A** |
| --- | --- | --- | --- | --- | --- | --- | --- | --- |
| The tobacco treatment program has measurable process components, outcomes, and metrics. |  |  |  |  |  |  |  |  |
| Evaluation and monitoring of the tobacco treatment program are reviewed on a consistent basis. |  |  |  |  |  |  |  |  |
| The tobacco treatment program has clear documentation to guide process and outcome evaluation. |  |  |  |  |  |  |  |  |
| The tobacco treatment program’s monitoring, evaluation, and outcomes data are routinely reported to the clinical care team. |  |  |  |  |  |  |  |  |
| The tobacco treatment program process components, outcomes, and metrics are easily assessed and audited. |  |  |  |  |  |  |  |  |

*Implementation & Training: Using processes that guide the direction, goals and strategies of the practice*

|  | **1- To little or no extent** | **2** | **3** | **4** | **5** | **6** | **7- To a very great extent** | **N/A** |
| --- | --- | --- | --- | --- | --- | --- | --- | --- |
| The tobacco treatment program clearly outlines roles and responsibilities for all staff. |  |  |  |  |  |  |  |  |
| The reason for the tobacco treatment program is clearly communicated to and understood by all staff. |  |  |  |  |  |  |  |  |
| Staff receive ongoing coaching, feedback, and training. |  |  |  |  |  |  |  |  |
| Program implementation is guided by feedback from stakeholders. |  |  |  |  |  |  |  |  |
| The tobacco treatment program has ongoing education across professions. |  |  |  |  |  |  |  |  |

*Outcomes & Effectiveness: Understanding and measuring practice outcomes and impact*

|  | **1- To little or no extent** | **2** | **3** | **4** | **5** | **6** | **7- To a very great extent** | **N/A** |
| --- | --- | --- | --- | --- | --- | --- | --- | --- |
| The tobacco treatment program has evidence of beneficial outcomes. |  |  |  |  |  |  |  |  |
| The tobacco treatment program is associated with improvement in patient outcomes that are clinically meaningful. |  |  |  |  |  |  |  |  |
| The tobacco treatment program is clearly linked to positive health or clinical outcomes. |  |  |  |  |  |  |  |  |
| The tobacco treatment program is cost-effective. |  |  |  |  |  |  |  |  |
| The tobacco treatment program has clear advantages over alternatives. |  |  |  |  |  |  |  |  |

*Workflow Integration: Designing the practice to fit into existing practices and technologies*

|  | **1- To little or no extent** | **2** | **3** | **4** | **5** | **6** | **7- To a very great extent** | **N/A** |
| --- | --- | --- | --- | --- | --- | --- | --- | --- |
| The tobacco treatment program is built into the clinical workflow. |  |  |  |  |  |  |  |  |
| The tobacco treatment program is easy for clinicians to use. |  |  |  |  |  |  |  |  |
| The tobacco treatment program integrates well with established clinical practices. |  |  |  |  |  |  |  |  |
| The tobacco treatment program aligns well with other clinical systems (e.g., EMR). |  |  |  |  |  |  |  |  |
| The tobacco treatment program is designed to be used consistently. |  |  |  |  |  |  |  |  |

*Organizational Readiness: Having the internal support and resources needed to effectively manage the practice*

|  | **1- To little or no extent** | **2** | **3** | **4** | **5** | **6** | **7- To a very great extent** | **N/A** |
| --- | --- | --- | --- | --- | --- | --- | --- | --- |
| Organizational systems are in place to support the various needs of the tobacco treatment program. |  |  |  |  |  |  |  |  |
| The tobacco treatment program fits in well with the culture of the team. |  |  |  |  |  |  |  |  |
| The tobacco treatment program has feasible and sufficient resources (e.g., time, space, funding) to achieve its goals. |  |  |  |  |  |  |  |  |
| The tobacco treatment program has adequate staff to achieve its goals. |  |  |  |  |  |  |  |  |
| The tobacco treatment program is well integrated into the operations of the organization. |  |  |  |  |  |  |  |  |

*Funding Stability: Establishing a consistent financial base for your program*

|  | **1- To little or no extent** | **2** | **3** | **4** | **5** | **6** | **7- To a very great extent** | **N/A** |
| --- | --- | --- | --- | --- | --- | --- | --- | --- |
| The tobacco treatment program exists in a in a US state that is fiscally supportive of tobacco treatment |  |  |  |  |  |  |  |  |
| The program exists in a health system that is fiscally supportive of tobacco treatment. |  |  |  |  |  |  |  |  |
| The tobacco treatment program is funded through a variety of sources. |  |  |  |  |  |  |  |  |
| The tobacco treatment program has a combination of stable and flexible funding. |  |  |  |  |  |  |  |  |
| The tobacco treatment program has sustained funding. |  |  |  |  |  |  |  |  |

*Communications: Strategic communication with stakeholders and the public about your program*

|  | **1- To little or no extent** | **2** | **3** | **4** | **5** | **6** | **7- To a very great extent** | **N/A** |
| --- | --- | --- | --- | --- | --- | --- | --- | --- |
| The tobacco treatment program has communication strategies to secure and maintain organizational support. |  |  |  |  |  |  |  |  |
| Tobacco treatment program staff communicate the need for the program to the organization. |  |  |  |  |  |  |  |  |
| The tobacco treatment program is marketed in a way that generates interest. |  |  |  |  |  |  |  |  |
| The tobacco treatment program increases organizational awareness of tobacco cessation |  |  |  |  |  |  |  |  |
| The tobacco treatment program demonstrates its value to the organization. |  |  |  |  |  |  |  |  |

*Program Adaptation: Taking actions that adapt your program to ensure its ongoing effectiveness*

|  | **1- To little or no extent** | **2** | **3** | **4** | **5** | **6** | **7- To a very great extent** | **N/A** |
| --- | --- | --- | --- | --- | --- | --- | --- | --- |
| The tobacco treatment program periodically reviews the evidence base. |  |  |  |  |  |  |  |  |
| The tobacco treatment program adapts strategies as needed. |  |  |  |  |  |  |  |  |
| The tobacco treatment program adapts to new science. |  |  |  |  |  |  |  |  |
| The tobacco treatment program proactively adapts to changes in the environment. |  |  |  |  |  |  |  |  |
| The tobacco treatment program makes decisions about which components are ineffective and should not continue. |  |  |  |  |  |  |  |  |

*Strategic Planning: Using processes that guide your program’s direction, goals, and strategies*

|  | **1- To little or no extent** | **2** | **3** | **4** | **5** | **6** | **7- To a very great extent** | **N/A** |
| --- | --- | --- | --- | --- | --- | --- | --- | --- |
| The tobacco treatment program plans for future resource needs. |  |  |  |  |  |  |  |  |
| The tobacco treatment program has a long-term financial plan. |  |  |  |  |  |  |  |  |
| The tobacco treatment program has a sustainability plan. |  |  |  |  |  |  |  |  |
| The tobacco treatment program’s goals are understood by all stakeholders. |  |  |  |  |  |  |  |  |
| The tobacco treatment program clearly outlines roles and responsibilities for all stakeholders. |  |  |  |  |  |  |  |  |

## **IMPLEMENTATION STRATEGIES**

**To what extent is your Tobacco Treatment Program using the following strategies?** **Select the response that best describes your current use.**

*Engaged Staff & Leadership*

| **Strategy** | **No longer maintaining** | **Maintaining use** | **Preparing to use** | **Never used** |
| --- | --- | --- | --- | --- |
| Secure leadership buy-in for the program. |  |  |  |  |
| Meet regularly with key leaders and clinic champions together and separately. |  |  |  |  |
| Identify and prepare clinic champions across different clinical care sites, including physicians, nurses, and tobacco treatment specialists. |  |  |  |  |
| Involve TTP leadership and staff in various multidisciplinary meetings (e.g., huddles, rounds, case conferences, tumor boards). |  |  |  |  |
| Share high impact articles and monographs on TTPs and smoking cessation with leaders. |  |  |  |  |

*Engaged Stakeholders*

| **Strategy** | **No longer maintaining** | **Maintaining use** | **Preparing to use** | **Never used** |
| --- | --- | --- | --- | --- |
| Collaborate with lung cancer and cancer screening programs. |  |  |  |  |
| Engage the Information Technology team. |  |  |  |  |
| Use internal marketing and communication to engage at the clinic or health system-level (e.g. staff newsletters, patient education materials). |  |  |  |  |
| Use external marketing and communication to engage at the state-level (e.g. to ensure that tobacco treatment is a goal of the state cancer control program). |  |  |  |  |

*Organizational Readiness*

| **Strategy** | **No longer maintaining** | **Maintaining use** | **Preparing to use** | **Never used** |
| --- | --- | --- | --- | --- |
| Develop a tobacco treatment program (TTP) manual to ensure continuity through staff turnover (e.g. include programmatic standards, reporting procedures, key performance indicators, etc.) |  |  |  |  |
| Define readiness (e.g., how much funding is needed, how much staff time is needed). |  |  |  |  |
| Conduct needs assessments. |  |  |  |  |

*Workflow Integration*

| **Strategy** | **No longer maintaining** | **Maintaining use** | **Preparing to use** | **Never used** |
| --- | --- | --- | --- | --- |
| Automate processes in electronic health records to screen, refer, or follow up with patients. |  |  |  |  |

*Implementation and Training*

| **Strategy** | **No longer maintaining** | **Maintaining use** | **Preparing to use** | **Never used** |
| --- | --- | --- | --- | --- |
| Utilize educational materials and communications across institutions. |  |  |  |  |
| Create a library of provider- and patient-facing educational resources. |  |  |  |  |

*Monitoring and Evaluation*

| **Strategy** | **No longer maintaining** | **Maintaining use** | **Preparing to use** | **Never used** |
| --- | --- | --- | --- | --- |
| Budget the staffing and resources necessary to monitor and evaluate. |  |  |  |  |
| Send regular monitoring and evaluation reports based on the metrics that matter to leaders and clinic champions. |  |  |  |  |

*Outcomes & Effectiveness*

| **Strategy** | **No longer maintaining** | **Maintaining use** | **Preparing to use** | **Never used** |
| --- | --- | --- | --- | --- |
| Use metrics and reporting requirements that align with leadership, clinical, research, and health system priorities. |  |  |  |  |
| Identify outcomes valued by external stakeholders, including patients, policy makers, and funders. |  |  |  |  |
| Design and adapt data collection and outcome reporting tools for tobacco treatment. |  |  |  |  |

## **FINANCING STRATEGIES**

1. Please answer the following questions related to the operational budget of the Tobacco Treatment Program (TTP). (Please round to the nearest $1,000.)
2. What has been the highest annual budget for your TTP?
   1. < $50,000
   2. $50,000-$100,000
   3. $100,000 - $250,000
   4. $250,000 - $500,000
   5. > $500,000
3. What is the optimal annual budget for your TTP?
   1. < $50,000
   2. $50,000-$100,000
   3. $100,000 - $250,000
   4. $250,000 - $500,000
   5. > $500,000
4. In 2023 (or its final year), how much additional funding do you think was needed to fully support your program and reach/treat patients?
   1. < $50,000
   2. $50,000-$100,000
   3. $100,000 - $250,000
   4. $250,000 - $500,000
   5. > $500,000
5. For the current TTP operational budget year, estimate the percentage of revenue received from each of the following sources (must add to 100%):

| Fee for service reimbursement | _____% |
| --- | --- |
| Bundled or episode-based payments | _____% |
| Institutional support | _____% |
| Grant funding | _____% |
| Charitable philanthropic donations (e.g., NRT) | _____% |
| Other, specify _________ | _____% |

1. Which providers in your health system bill for fee for service reimbursement for tobacco treatment services? Select all that apply.
   1. Psychologist
   2. Tobacco treatment specialist
   3. Pharmacist
   4. Physician
   5. Social Worker
   6. Advanced Practice Provider (Nurse Practitioner, Physician Assistant)
   7. Other __________________
2. What services or personnel are **not** funded through the operational TTP budget but support the TTP? Select all that apply.
3. Reporting
4. IT services
5. Faculty
6. TTP staff
7. Marketing and Communications
8. Dedicated Office Space
9. Equipment
   1. Other: _____________________

## **OUTCOMES**

**Select the choice that best describes your current monitoring of the following tobacco treatment program (TTP) metrics:**

1. Tobacco Use
   1. We continue to assess tobacco use status (i.e., cancer patients screened for tobacco use, among all cancer patients).
   2. We assess tobacco use status using a different format.
   3. We do not currently assess tobacco use status.
2. TTP Referral
   1. We continue to assess TTP referrals (i.e., smokers referred to tobacco treatment, among all cancer patients who smoke).
   2. We do not currently assess TTP referrals.
   3. We assess TTP referrals using a different format.
3. Reach
   1. We continue to use the C3I definition to assess TTP reach (i.e., patients receiving tobacco treatment, among all cancer patients who smoke).
   2. We assess TTP reach using a different format.
   3. We do not currently assess TTP reach.
4. Effectiveness
   1. We continue to use the C3I definition to assess TTP effectiveness (i.e., patients who achieve abstinence at 6 months, among all cancer patients enrolled).
   2. We assess TTP effectiveness using a different format.
   3. We do not currently assess TTP effectiveness.

**Please describe the implementation and expansion of your tobacco treatment by entering the number of clinics the TTP was implemented in in each of these settings, at each of these time points.**

| **Settings** | **How many clinics piloted your program (in the first year of C3I funding)?** | **How many clinics is your program currently implemented in?** | **How many clinics do you expect to be in one year from now?** |
| --- | --- | --- | --- |
| Medical Oncology clinic (within your health system) |  |  |  |
| Radiation Oncology (within your health system) |  |  |  |
| Surgical Oncology (within your health system) |  |  |  |
| Primary care clinics (within your healthcare system) |  |  |  |
| Non-oncology and specialty clinics (within your health system) |  |  |  |
| Affiliated clinics and health systems (e.g., regional hospitals) External Settings |  |  |  |
| Other: ________________ |  |  |  |

## **SUSTAINMENT OF CORE COMPONENTS/FUNCTIONS**

**Please indicate the current status of these activities/components as it relates to your Tobacco Treatment Program.**

|  | **Never needed** | **No longer maintained** | **Decreasing** | **Increasing** | **Maintaining** |
| --- | --- | --- | --- | --- | --- |
| Cessation medications provided or covered by the program |  |  |  |  |  |
| Staff education and training about the TTP (include frequency) |  |  |  |  |  |
| Technology services (e.g., Interactive Voice Response) |  |  |  |  |  |
| Equipment (e.g., computers) |  |  |  |  |  |
| Office Space |  |  |  |  |  |
| Resources related to EHR modifications including access to EHR IT experts |  |  |  |  |  |
| Patient recruitment support |  |  |  |  |  |
| Community outreach and engagement |  |  |  |  |  |
| Marketing and communications |  |  |  |  |  |
| Reporting to monitor program outcomes |  |  |  |  |  |

###

**Please indicate the degree to which you perceive each of the following partners to be engaged in the tobacco treatment program.**

|  | **No extent (1)** | **(2)** | **(3)** | **(4)** | **(5)** | **(6)** | **Full extent (7)** | **NA** |
| --- | --- | --- | --- | --- | --- | --- | --- | --- |
| Patient and caregiver advisory groups |  |  |  |  |  |  |  |  |
| Community oncology network leadership |  |  |  |  |  |  |  |  |
| Pharmacy |  |  |  |  |  |  |  |  |
| Clinicians and staff who implement the program (e.g., nurses, physicians, psychologists, tobacco treatment specialists) |  |  |  |  |  |  |  |  |
| Department in which the program is housed |  |  |  |  |  |  |  |  |
| Cancer center leadership |  |  |  |  |  |  |  |  |
| Clinical Center of Excellence (clinical entity that oversees cancer care) |  |  |  |  |  |  |  |  |
| Clinical Council (translates national guidelines into local practice) |  |  |  |  |  |  |  |  |
| Chief Executive Officer (CEO) of Health System |  |  |  |  |  |  |  |  |
| Chief Financial Officer (CFO) of Health System |  |  |  |  |  |  |  |  |
| Chief Medical Officer (CMO) of Health System |  |  |  |  |  |  |  |  |
| Chief Information Officer (CIO) of Health System |  |  |  |  |  |  |  |  |
| Chief Nursing Officer of Health System |  |  |  |  |  |  |  |  |
| Chief Quality Officer of Health System |  |  |  |  |  |  |  |  |
| Academic Dean (e.g., Medical School) |  |  |  |  |  |  |  |  |
| Health System Board of Directors |  |  |  |  |  |  |  |  |
| Other: _____________________________ |  |  |  |  |  |  |  |  |
